# Flow Injection Analysis: a new tool to automate extraction processes

**DOI:** 10.1155/S1463924686000135

**Published:** 1986

**Authors:** M. D. Luque de Castro

**Affiliations:** Department of Analytical Chemistry Faculty of Sciences University of Córdoba Córdoba Spain


Journal of Automatic Chemistry/*Journal of Clinical Laboratory Automation*, Vol. 8, No. 2 (April–June 1986), pp. 56–62

### Flow Injection Analysis: a new tool to automate extraction processes

**M. D. Luque de Castro**
*Department of Analytical Chemistry, Faculty of Sciences, University of Córdoba, Córdoba, Spain*

Present needs have forced the growth and improvement of analytical methods; an area of analytical chemistry which has received a great deal of attention over the last few decades is automatic analysis, with many and varied instruments having been developed and commercialized.

The most important automatic methods can be classed into three groups [1–3]: (1) *Discrete or batch*, in which the sample preserves its identity in a reaction cup, detection being performed discontinuously. (2) *Continuous methods*, which involve the constant circulation of stream through the system wherein the sample is introduced by aspiration or injection and subsequently led to the detection cell. The methods in this group can be further classified according to whether they use air bubbles (SFA) [4 and 5] or not UFA to avoid carry-over between successively injected samples. Unsegmented flow methods can be divided into those injecting FIA [3 and 6] or aspirating (CCFA) [7] the sample into the system. (3) *Robotic methods*, which mimic the actions of an operator and are still in early stages of development [8 and 9].

Owing to the complexity of the samples usually analysed by automatic methods (in clinical and environmental work particularly) it is most often necessary to employ separation techniques in order to improve the selectivity (and sensitivity in many cases) of these determinative methods.

Flow Injection Analysis (FIA) is a relatively recent technique [3 and 6] which, because of its peculiar features (simplicity, rapidity, economy and versatility), meets present requirements; hence the growing interest in methods developed by this technique.

FIA allows the incorporation into its configurations of very different separation techniques, which can be classified according to the phases involved (gas–liquid, gas–solid, liquid–solid and liquid–liquid) to solve the various problems encountered in analytical chemistry. Of these systems, it is dialysis (especially in the clinical chemistry field [10]) and liquid–liquid extraction [11] which have been most extensively used in conjunction with FIA.

### Features of the FIA/liquid–liquid extraction binomial

The on-line incorporation of a liquid–liquid extraction system into an FIA configuration was simultaneously suggested by Karlberg and Thelander [12] and Bergamin *et al.* [13] in 1978. In both cases, the extraction system was located behind the injection valve. Currently, this separation unit can be placed in the original position, or prior to the injection system, in a such manner that the separation process results in a continuous stream of organic phase containing the analyte, which fills the loop of the injection valve [14 and 15]. Less frequent in FIA is the off-line incorporation of the extraction system [16 and 17]. This coupling has evolved in that configurations of varying complexity are available according to particular needs. The following types can be distinguished:

(1) *Without phase separation*, which is the simplest mode and in which the aqueous sample is injected into a single-channel configuration enclosing the organic stream extractant, which flows through the extraction coil. This is where the formation of an extractable complex between the analyte and the reagent dissolved in the organic phase, which is measured as it passes through the fluorimetric cell [18], takes place.

(2) *Single extraction*, with the separation system located on or off-line.

(3) *Multi-extraction*, in which the separation process is repeated several times by using the same or a different extractant in the successive stages [19 and 20] thus increasing the selectivity, sensitivity and efficiency of the overall process.

(4) *Back extraction*, which is a multi-stage extraction mode in which the aqueous sample is first extracted into an organic medium and then back-extracted into an aqueous phase, where measurements are performed [21].

The presence of an organic phase within an FIA system involves taking a series of special precautions because of its corrosive properties. The transport tubing, connectors and extraction system must be steel, platinum, glass or Teflon. An organic solvent stream can be created (*a*) by means of a peristaltic pump the PVC flexible tubes commonly used are useless, so that to tubes of an inert material have to be employed—modified PVC, silicon rubber, fluoroplast for example; or (*b*) by the displacement technique, which involves pumping an aqueous stream into a closed container (use of a peristaltic pump with ordinary tubing). The container is filled with organic solvent, which is fed at a constant flow-rate towards the FIA system. Or (*c*) by setting a constant pressure with the aid of an inert gas which forces the organic extractant to circulate along the FIA configuration.

The most serious shortcoming currently involved in the use of liquid–liquid extraction/FIA is a lack of work on its theory. The studies performed in this area have been only partial, although the number of papers reporting new contributions in this area is increasing [22–25].

The laser-induced excitation technique means that the transient concentration profile in the narrow reaction tube can be measured directly at different times during the process and for different chemical and hydrodynamic characteristics of the system. The results obtained with a single chemical system do not allow any generalizations [26].

In their paper on the hydrodynamic and interfacial origin of phase segmentation [24], Sweileh and Cantwell developed a semi-quantitative physicochemical model for the process by which alternating segments of aqueous and immiscible organic phases are produced at the confluence streams of the two liquid. A growing 'drop' of one phase is dislodged to produce a segment when the hydrodynamic force exerted on it as a result of the flow of the other solvent is equal to the interfacial force holding it in place. Hydrodynamic forces are expressed by Poiseuille's and Bernouill's laws, while the interfacial force is expressed by a form of the Tate equation [27 and 28] in terms of liquid–liquid interfacial tension and solid–liquid–liquid contact angle. These authors have also derived a series of equations for calculating:

(i) Extracted analyte fraction, Ø, as a function of distribution ratio, $D$, and the flow-rate ratio of organic phase to aqueous phase, $F_o/F_a$ : $Ø = D(F_o/F_a)/(1 + D)(F_o/F_a)$.

(ii) Dependency of the peak area, $A$, on the flow-rates used, the distribution ratio and a proportionality constant characteristic of the chemical system: $1/A = (F_t + F_o(1 - D))/nDK$ where ($F_t$ is overall flow-rate and, $n$ the number of injected phase mol), which is indicative of linearity and zero intercept for the plot $1/A$ versus $(F_t + F_o(D - 1))$, an equation which has been tested for caffeine, by changing $F_o$ over a wide range (2.7 to 8.2 ml/min) [25]. The equation becomes more complicated when a dual membrane is used to perform measurements in the organic and aqueous separated phases [29].

(iii) Calculation of the peak height in an extraction/FIA/AAS system as a function of the analyte concentration in the injected sample, $C$, flow-rate ratios, extracted analyte fraction, sensitivity of the spectrophotometer, $S_f$, and a factor, $R_v$, similar to that of the dispersion described by Ruzicka [6], adapted to systems incorporating liquid–liquid extraction. The equation has the form:

$$\text{Peak height} = C.S_f.R_v \frac{F_{\text{carrier}}}{F_o} Ø \frac{F_M}{F_M + F_c}$$

($F_{\text{carrier}}$ being the flow-rate of the aqueous solution into which the sample in injected, $F_M$ the flow-rate of the pure organic phase to the separator exit and $F_c$ the flow-rate of the pure organic solvent which is mixed with the sample to compensate for the disparity between $F_M$ and the velocity of the nebulizer) [23 and 25].

The efficiency of a separation process has been studied by Rossi *et al.* [20], who have determined the most suitable characteristics of the extraction unit (coil) as a function of the phases involved, and the influence of FIA variables (such as flow-rate, injected volume and travel time) on the efficiency.

A recent paper by Nord and Karlberg [22] deals with the mechanism of dispersion in flow injection extraction systems, establishing the influence of this factor on the peak-width as a function of the variables originating the dispersion phenomenon.

### Advantages of FIA/extraction systems

The characteristic versatility of the FIA technique means that configurations can be adapted to the features of the chemical system involved in the process (for example streams merging with the two-phase system to favour the extraction process through a salting-out effect [13], changes in viscosity, pH scanning [30]). The kinetic character of this technique (measurements made in a non-equilibrium state, which allow classing FIA methods as fixed-time kinetic methods [3]), is increased in the FIA/liquid–liquid extraction conjunction; this results in a significant increase in the inherently high selectivity of FIA [31 and 32]. The advantages of an FIA/liquid–liquid extraction system over such automatic methods as segmented ones can be summarized as follows: (1) lower sample, reagent and organic solvent consumption; (2) faster determinations; (3) simpler assemblies; (4) greater reproducibility; (5) lower cost of the instrumentation; (6) possibility to work out contact with atmosphere, and diminished exposure to organic solvent vapours, characteristic of continuous automatic systems.

### Components of an FIA liquid–liquid extraction system

Three essential components are necessary in an FIA automatic solvent extraction system to develop the three physical operations involved in the overall process: (1) phase segmentation; (2) extraction and (3) phase separation. Phase segmentation involves dividing the aqueous and organic phases into alternating segments. The extraction process requires that the segmented phases be allowed to remain in contact, while the analyte approaches a state of thermodynamic equilibrium, by partitioning between the two phases. This process is achieved when the segmented phases are pumped through an extraction coil. Finally, the phase separation process entails the partitioning of the segmented phases. During this process, the unwanted phase is wasted while the other one is 'resampled' or pumped through for detection. Thus, the basic components required are solvent segmentor; an extraction coil, and a phase separator. Figure 1 shows how these elements are assembled in the system.

The segmentor is the unit in which the streams of the two phases involved merge and is intended to obtain identical alternate segments of both immiscible liquids attaining the extraction coil. The simplest varieties of this component are the confluence points show in figure 2 (90–90 T, 30–30 Y, and 45–45 W). The confluence of these three types of segmentors has been studied by Kawase [17],

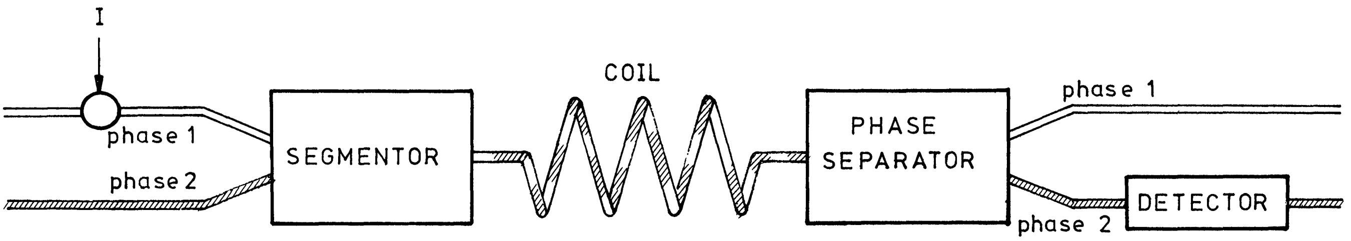

*Figure 1. General arrangement of a continuous liquid–liquid extraction system.*

using capillaries with different inner diameters. When the extraction coil has a large inner diameter (1 mm), the three configurations yield identical results, while with a small diameter (0.5 mm) the T and Y configurations provide larger segments than the W one. The type of segmentor most frequently used is a simple modification of the A-8 and A-10 Technicon connectors (figure 2) and allows the segments of both phases to be controlled. It consists of a tube with three openings. The aqueous phase goes in through a platinum capillary, which is perpendicular to the former. Two adjustable, concentric Teflon tubes shut off the tube at the other opening in the same direction as the aqueous phase. The height of the inner tube can be adjusted to that of the perpendicular opening. The length of the segments coming out of the main conduit of this tube depends on the height; the inner volume of the mixing chamber; and the flowrates of both phases or their ratio, which is equivalent to the volume ratio in a discontinuous liquid–liquid extraction system (this generally approximates to unity). The most common situation is to have identical segments, about 5 mm in length.

The transfer of matter between the segments of both phases is carried out in the extraction coil. When the walls are Teflon, the aqueous phase, excluded or repelled by the walls, is carried as bubbles; when the walls are glass, the aqueous phase wets the walls and the organic phase is transported as bubbles by the aqueous phase. Shelly *et al.* [19] have established some criteria for selecting the coil. Thus, its constituent material must allow the analyte to pass to the bubble phase. In addition, the ratio between the interphase area and the initial analyte volume must be as high as possible, and the analyte movement should be facilitated to achieve maximum efficiency.

The phase separator receives the segmented flow from the coil and continuously splits it into two separate flows in both phases. The efficiency of the separation is seldom quantitative: it usually ranges between 80 and 95%. The phase in which the determination is to be carried out should be kept completely free from the other in order that, designing the phase separator, one must take into account that the inner volume should be as small as possible in order to avoid parasitic dispersion phenomena. These, in addition to diluting the sample, will yield a weaker signal, resulting in a loss of reproducibility.

There are three main types of continuous separator (figure 3). (1) Devices using a chamber relying on gravity to separate the phases. The heaviest phase comes out of the bottom part and the least dense out of the top one. Figure 3(*a*) shows an early separator of this type, described by Bergamin *et al.* [13]. More recently, the Burgueras have designed a more sophisticated model which has yielded very good results [33].
(2) Devices with a T-separator, using gravity with or without a sort of phase guide made of a material wetted by one phase, but not by the other [19]. Figure 3(*b*) shows a separator with a Teflon piece stuck to the inside which helps to separate the less dense organic phase up to the exit. Strips of hydrophobic paper can also be used with this purpose.

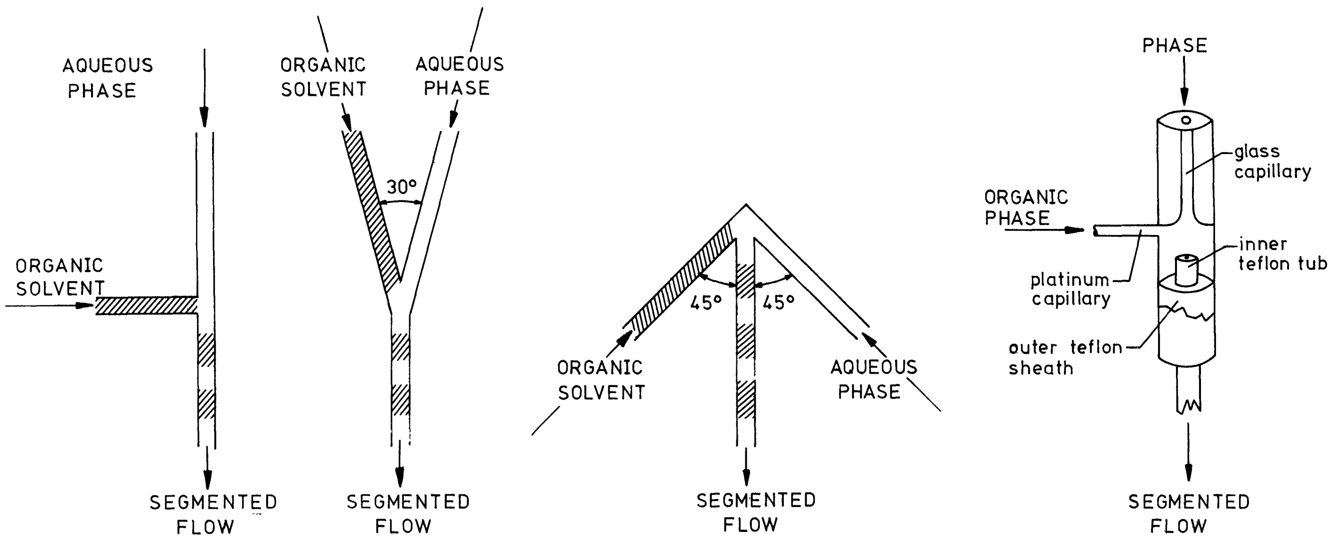

*Figure 2. General types of solvent segmentors.*

(3) Devices with a membrane phase separator based on the selective permeability of a microporous membrane (0.7–0.9 μ of pore diameter) towards the phase which wets the membrane material. The organic phase which goes through it is free from any aqueous phase. Several configurations have been designed [17, 26, 29, 34 and 35], although there are no significant differences between them. Modified versions of this unit incorporate a supported Teflon membrane providing better results [36] or a dual-membrane separator with lipo and hydrophylic membranes which generate clean, aqueous and organic phases which can pass through different detection systems. Is in these cases, a third exit channel wastes the small portion of mixture which has crossed no membranes [29].

The membrane phase separator has the following advantages as compared to other types of continuous separator. Firstly, it has a smaller inner volume, which lessens the dispersion or dilution of the analyte or its reaction product, which results in lesser band broadening and higher sensitivity. Secondly, it is more reliable for separating the phases at higher flow-rates and thus its use shortens the analysis time. Thirdly, it can be used with a variety of water-immiscible solvents because no density difference between the aqueous and organic phases is required. Fourthly, the separation efficiency is greater than for other separating devices (above 90–95%).

### Applications

Automated FIA/liquid extraction systems have helped the solution of analytical problems, especially in clinical, environmental and pharmaceutical chemistry (see table 1), the table also summarized the most significant features of the suggested methods. These applications have been systematized according to the analyte determined (inorganic and organic compounds) and, within each of these groups, depending on the detection system used.

### Determination of organic compounds

Photometric detection has been frequently used in FIA [3], and the technique can be associated with continuous extraction. the first method developed for the determination of an inorganic species (molybdenum in soils) used a phase-separation chamber such as that shown in figure 3(*a*), which was also employed by Klinghoffer *et al.* for the environmental and industrial determination of cadmium and lead with ditizone [30]. In this latter case, the

*Table 1. Features of the automatic FIA extraction methods.*

| Analyte | Chemical foundation | Organic phase | Separating system | Detection system |
|---|---|---|---|---|
| Mo | CF | Isoamyl alcohol | Gradient chamber | Photometric |
| Pb, Cd | | Carbon tetrachloride | DOH connector-paper phase | |
| Cd | | Chloroform | Paper phase | |
| $PO_4^{3-}$ | | Isobuthyl acetate | Membrane | |
| U | | Tributil phosphate/ heptane | Modified T-piece | |
| K | IPF | 1,2-dichloro-ethane | No | Fluorimetric |
| Ga | CF | Isoamyl alcohol | Membrane | |
| Cu, Ni, Zn, Pb | | 4-methyl-2-pentanone | Membrane | AAS |
| Zn | | | | |
| Zn | | | Membrane | |
| $ClO_4^-$ | IPF | | Glass-Teflon T-piece | |
| $NO_3^-$-$NO_2^-$ | | | | |
| $NO_3^-$, $NO_2^-$ | | | | |
| Caffeine | NCR | Chloroform | Teflon T-piece | Photometric |
| Codeine | IPF | | | |
| Anionic surfactans | | | gradient chamber | |
| Anionic surfactans | | Toluene | | |
| Cationic surfactans | | chloroform | Y-connector | |
| Procyclidine | | | Membrane | |
| Diphenyldramamine | NCR | Cyclohexane | Paper phase-teflon membranes | |
| 8-chlorotheofilline | | | | |
| Vitamin $B_1$ | RR | Chloroform | Teflon T-piece | Fluorimetric |
| Steroids | IPF | 1,2-dichloro-ethane | | Chemiluminescence |

FC = complex formation
IPF = ion-pair formation
NCR = non-chemical reaction
RR = redox reaction
dl = determination limit
dd.l = detection limit
(a) = manual injection
(b) = automated injection
ppM = parts per milliard.

optimization was carried out by incorporating into the sample carrier container an autoburette which allowed a pH gradient to be formed in the aqueous solution, thus permitting the fast determination of the influence and optimum value of this variable. This pH-scanning method has been used in several cases by the author for different applications [6]. The determination of cadmium in urine by the above reaction has been proposed by the Burgueras [33], who used a new design of phase separator based on density differences. The fully automated determination of uranium in ore leachates improves significantly upon previously reported procedures using segmented flow systems in terms of speed and sensitivity [37]. The most significant features of the method used to analyse orthophosphate is seminal plasma and urine without deproteinization [38], are summarized in table 1.

A very simple configuration containing no separation unit is utilized for the fluorimetric determination of potassium by complexation with a macrocylic compound extractable into aniline-naphtalene sulphonate [18]. The method involves injecting the aqueous sample solution into a stream of 1,2-dichloroethane, which is carried to the detector through a mixing coil where the extraction process is accomplished. The turbidity of the solution in the flow-cell poses no irreproducibility problems. The fluorimetric determination of gallium was exploited by Imasaka *et al.* [26] to design a membrane separator and study transient phenomena by a laser-excitation technique.

The configuration including an extraction system prior to the injection valve (figure 4) is the most commonly used in the extraction/FIA/AAS association for the preconcentration and separation of the analyte. The advantages of this triple conjunction have been noted by several authors [3, 6 and 11]; for example the determination of copper, nickel, zinc and cadmium proposed by Nord and Karlberg [39], zinc in biological and environmental samples [40] and iron matrices [23], and the indirect determination of perchlorate in serum and urine, based on the ion-pair formation between this anion and the copper(I)/6-methylpicolinaldehyde azine complex [15]. The sequential determination of nitrate and nitrite in food is an example of the unexploited possibilities of this association in simultaneous analysis. The method is based on the formation of an ion-pair between nitrate and the Cu[I]-neocuproine complex which is extracted into MIBK. The samples contain an oxidant (Ce[IV]) or inhibitor (amidosulphonic acid) for the determination of the sum of both anions and nitrate alone, respectively [41]. The same chemical reaction, in the absence of redox and inhibitor agents permits the individual determination of these anions [42].

*Table 1 continued.*

| Injected volume (μl) | Determination range | Sampling frequency ($h^{-1}$) | Field of application | Other features | Ref. |
|---|---|---|---|---|---|
| 2 ml | d.l. 0.05 ppm | 30 | Agricultural | | 13 |
| 30, 100 | 200–1000 ppM | 90 | Industrial | pH scanning | 30 |
| 90 | d.l. 12 ppb | | Clinical | new phase separator | 33 |
| | 0.25–2.5 ppm | 80 | Clinical | | 38 |
| 100 | d.l. 0.1 ppm | 50 | Industrial | | 18 |
| 10 | $2 \times 10^{-4}$–$10 \times 10^{-4}$M | | | excitation laser technique | 27 |
| | $4 \times 10^{-6}$–$8 \times 10^{-4}$M | 40 | | | 39 |
| 110 | dd.l. 1 ppm | 40 | | | |
| 200 | 20–800 ppm | | Clinical | | 40 |
| 385 | dd.l. 0.2 ppm | | | gas pressure | 23 |
| 130 | 0.1–5.0 ppm | 45 | | | 15 |
| 130 | 0.13–2.20 ppm | 20 | Food | sequential determination | 41 |
| 130 | 0.1–2.2 ppm | | | | |
| | 0.5–10.0 ppm | 40 | | | 42 |
| 12–25 | | 100 | | | 12 |
| 25–40 | $0.5 \times 10^{-4}$–$9 \times 10^{-4}$M | 60 | | | 43 |
| | d.l. 1.25 mM | 80 | Industrial | new phase separator | 34 |
| 150 | 0.01–1.00 ppm | | Environmental | | 49 |
| 6 (a) | 0.3–3.0 mM (a) | | | | |
| 8 (b) | 0.1–1.0 mM (b) | | | prior extraction | 17 |
| 50 | $2.5 \times 10^{-5}$–$2 \times 10^{-3}$M | 240 | Pharmaceutical | gas pressure | 45 |
| | $0.25 \times 10^{-3}$–$2 \times 10^{-3}$M | 120 | Pharmaceutical | simultaneous measure- | |
| | | | | ments organic and | 29 |
| | | | | aqueous phases | 46 |
| 150 | | 70 | Pharmaceutical | | |
| 5–20 | 3–30 p mol | | Clinical | | 47 |

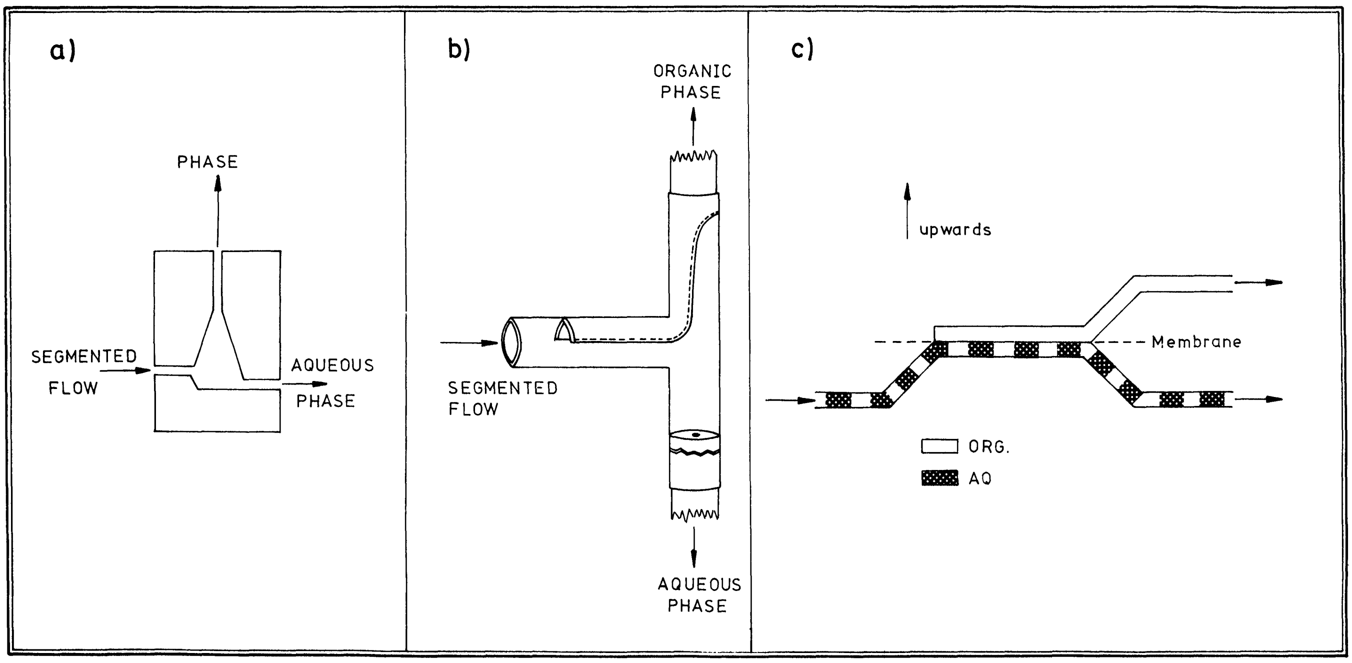

*Figure 3. Different types of phase separators.*

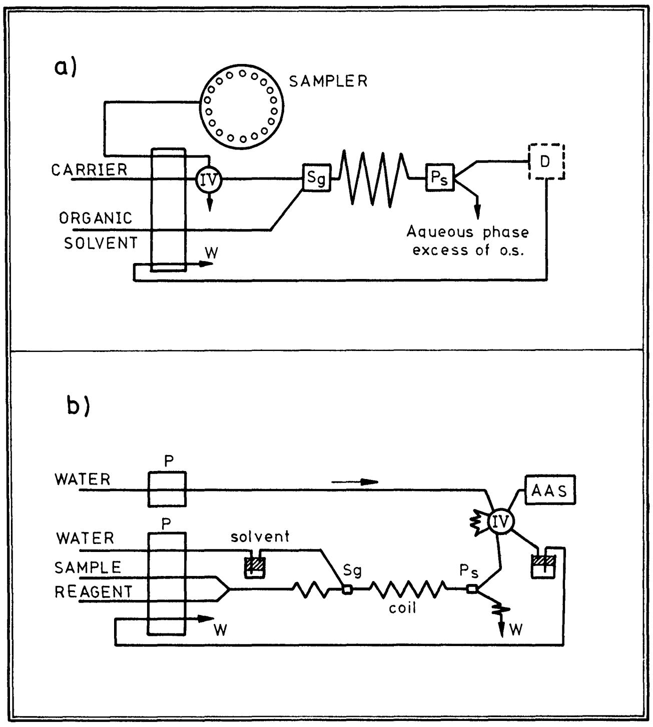

*Figure 4. General arrangements of the FIA/extraction systems. (a) FIA/extraction/molecular optical detector; (b) FIA/extraction/ AAS assembly.*

### Determination of organic compounds

This is the easiest and most convenient field of application of extraction techniques, because the affinity of the analyte to the second solvent reduces the complexity of the chemical reactions prior to or simultaneously with the separation process; it may even make them unnecessary. the ion-pair formation with a chromophoror or fluorophoror is the most usual chemical basis for these methods.

The first photometric method for determination of an organic compound (caffeine in pharmaceuticals) was reported by Karlberg *et al.*, who thus introduced the first FIA/extraction system and established the influence of generical variables of these systems. The sample, a pharmaceutical prepared from acetylsalicylic acid, was similar to that utilized by the same authors for determination of codeine with a prior ion-pair formation reaction [43]. The sample rate (table 1) and precision are better than 1% in both cases. The determination of anionic and cationic surfactants based on the ion-pair formation (table 1) represents an interesting contribution of the extraction/FIA/photometry association in industrial and environmental chemistry [17, 34 and 44], while in pharmaceutics methods have been developed for determination of procyclidine [45], and diphenyldramamine and 8-chlorotheophylline [29]. This last work is an interesting example of simultaneous determination: a dual membrane separator (lipophylic-Teflon- and hydrophylic-paper-) was used to obtain clear aqueous and organic phases, each of them being led to a different spectrophotometer. The aqueous portion, pH 10, contains 8-chlorotheophylline, while the organic one (cyclohexane) contains diphenyldramamine.

A fluorimetric method based on a redox reaction (thiamine/thiochrome) has been suggested for determination of vitamine B1 in pharmaceuticals [46]. There is only a single method involving chemiluminescence detection for the determination of steroids and bile sulfates with lucigenin which is also based on a redox reaction and subsequent ion-pair formation [47].

### Non-analytical applications of the liquid–liquid extraction in FIA

The extraction/FIA association has also been applied to non-analytical aspects, some of which have been commented in the consideration of the features of these systems (calculation of the extracted analyte fraction [23], the peak area [25] and height [23] as a function of the flow-rate and other characteristic parameters of the chemical system). Other contributions of this type include calculation of acidity constants, based on the use of a dual-membrane phase separator. The measurements performed in both phases require knowledge of the concentration of the anionic and neutral forms, through the expressions relating the peak area obtained for each phase, $A_a$ and $A_o$, to the proton activity, flow-rates, distribution coefficients etc., calculated in the same experiments [49].

Nevertheless, contributions in this area are still not very numerous, although the author predicts a greater development of these aspects aimed at the study of reaction mechanisms and kinetics with the aid of laser-induced excitation [26] or multi-extraction systems [19 and 20], and fast scan detectors [19].

### References

1. Foreman, J. K. and Stockwell, P. B. (Eds), *Automatic Chemical Analysis* (Ellis Horwood Ltd, Chichester, 1975).
2. Foreman, J. K. and Stockwell, P. B. (Eds), *Topics in Automatic Chemical Analysis* (Ellis Horwood Ltd, Chichester, 1979).
3. Valcárcel, M. and Luque de Castro, M. D., *Flow Injection Analysis: Principles and Applications* (Ellis Horwood Ltd, Chichester, 1985).
4. Furman, W. B., *Continuous Flow Analysis: Theory and Practice* (Marcel Dekker, New York, 1976).
5. Coakley, W. A., *Handbook of Automatic Analysis. Continuous Flow Techniques* (Marcel Dekker, New York, 1982).
6. Ruzicka, J. and Hansen, E. H., *Flow Injection Analysis* (Wiley and Sons, New York, 1981).
7. Goto, M., *Trends in Analytical Chemistry*, **2** (1983), 92.
8. Barker, P., *Computers in Analytical Chemistry* (Pergamon Press, Oxford, 1983).
9. Minski, M., *Robotics* (Doubleday & Co., New York, 1985), and Engelberger, J. R., *Robotics in Practice* (Kogan Page, London, 1980).
10. Linares, P., Luque de Castro, M. D. and Valcácel, M., *Critical Reviews in Analytical Chemistry*, **3** (1985), 225.
11. Karlberg, B. in *Chemical Derivatization in Analytical Chemistry Vol. 2: Separation and Continuous Flow Techniques* Eds Frei, R. W. and Lawrence, J. F. (Plenum Press, New York, 1982).
12. Karlberg, B. and Thelander, S., *Analytical Chimica Acta*, **98** (1978), 1.
13. Bergamin, H. F., Medeiros, J. X., Reis, B. F. and Zagatto, E. A. G., *Analytica Chimica Acta*, **101** (1978), 9.
14. Nord, L. and Karlberg, B., *Analytica Chimica Acta*, **125** (1981), 199.
15. Gallego, M. and Valcárcel, M., *Analytica Chimica Acta*, **169** (1985), 161.
16. Deratini, A. and Sebille, B., *Analytical Chemistry*, **53** (1981), 1742.
17. Kawase, J., *Analytical Chemistry*, **52** (1980), 2124.
18. Kina, K., Shiraishi and Ishibashi, N., *Talanta*, **25** (1978), 295.
19. Shelly, D. C., Rossi, T. M. and Warner, I. M., *Analytical Chemistry*, **54** (1982), 87.
20. Rossi, T. M., Shelly, D. C. and Warner, I. M., *Analytical Chemistry*, **54** (1982), 2056.
21. Bengtsson, M. and Johansson, G., *Analytical Chimica Acta*, **158** (1984), 147.
22. Nord, L. and Karlberg, B., *Analytica Chimica Acta*, **164** (1984), 233.
23. Sweileh, J. A. and Cantwell, F. F., *Analytical Chemistry*, **57** (1985), 420.
24. Cantwell, F. F. and Sweileh, J. A., *Analytical Chemistry*, **57** (1985), 329.
25. Sweileh, J. A. and Cantwell, *Canadian Journal of Chemistry* (in press).
26. Imasaka, T., Harada, T. and Ishibashi, N., *Analytica Chim-Acta*, **129** (1981), 195.
27. Davies, J. T. and Rideal, E. K., *Interfacial Phenomena*, 2nd edn (Academic Press, New York, 1963), Chapter 1.
28. Adamson, A. W., *Physical Chemistry of Surfaces*, 2nd edn (Interscience, New York, 1967), Chapters 1 and 7.
29. Fossey, L. and Cantwell, F. F., *Analytical Chemistry*, **55** (1983), 1882.
30. Klinghoffer, O., Ruzicka, J. and Hansen, E. H., *Talanta*, **27** (1980), 169.
31. Lázaro, F., Luque de Castro, M. D. and Valcárcel, M., *Analytica Chimica Acta*, **169** (1985), 132.
32. Linares, P., Luque de Castro, M. D. and Valcárcel, M., *Analytica Chimica Acta*, **161** (1984), 257.
33. Burguera, J. L. and Burguera, M., *Analytica Chimica Acta*, **153** (1983), 207.
34. Kawase, J., Nakae, A. and Yamanaka, M., *Analytical Chemistry*, **51** (1979), 1640.
35. Ogata, K., Taguchi, K. and Imanad, T., *Analytical Chemistry*, **54** (1982), 2127.
36. Backstrom, K., Danielsson, L. G. and Nord, L., *Analytica Chimica Acta*, **169** (1985), 43.
37. Lynch, T. P., Taylor, A. F. and Wilson, J. N., *The* Analyst, **108** (1983), 470.
38. Ogata, K., Taguchi, K. and Imanari, T., *Bunseki Kagaku*, **31** (1982).
39. Nord, L. and Karlberg, B., *Analytica Chimica Acta*, **145** (1983), 151.
40. Ogata, K., Tanabe, S. and Imanari, T., *Chemical Pharmaceutical Bulletin*, **31** (1983), 1419.
41. Gallego, M., Silva, M. and Valcárcel, M., *Analytica Chimica Acta* (in press).
42. Ibid. Fresenius Z. *Analytische Chemie* (in press).
43. Karlberg, B., Johansson, P. A. and Thelander, S., *Analytica Chimica Acta*, **104** (1979), 21.
44. Hirai, Y. and Tomokuni, K., *Analytica Chimica Acta*, **167** (1985), 409.
45. Fossey, L. and Cantwell, F. F., *Analytical Chemistry*, **54** (1982), 1693.
46. Karlberg, B. and Thelander, S., *Analytica Chimica Acta*, **114** (1980), 129.
47. Maeda, M. and Tsuji, A., *The Analyst*, **110** (1985), 665.
48. Johansson, P. A., Karlberg, B. and Thelander, S., *Analytica Chimica Acta*, **114** (1980), 215.
49. Fossey, L. and Cantwell, F. F., *Analytical Chemistry*, **57** (1985), 992.

